# Long-term results in malignant pleural mesothelioma treated with neoadjuvant chemotherapy, extrapleural pneumonectomy and intensity-modulated radiotherapy

**DOI:** 10.1186/s13014-015-0575-5

**Published:** 2015-12-30

**Authors:** Christian Thieke, Nils H. Nicolay, Florian Sterzing, Hans Hoffmann, Falk Roeder, Seyer Safi, Juergen Debus, Peter E. Huber

**Affiliations:** Department of Radiation Oncology, Heidelberg University Hospital, Heidelberg, Germany; CCU Radiation Oncology, German Cancer Research Center, Heidelberg, Germany; Department of Thoracic Surgery, Thoraxklinik, Heidelberg University Hospital, Heidelberg, Germany; CCU Molecular Radiation Oncology, German Cancer Research Center, Heidelberg, Germany; Present address: Department of Radiation Oncology, University of Munich (LMU), Marchioninistr. 15, 81377 Munich, Germany

**Keywords:** Malignant pleural mesothelioma, Trimodal therapy, Intensity modulated radiation therapy, Extrapleural pneumonectomy, Chemotherapy

## Abstract

**Introduction:**

We investigated the clinical outcome and the toxicity of trimodal therapy of malignant pleural mesothelioma (MPM) treated with neoadjuvant chemotherapy, extrapleural pneumonectomy (EPP) and adjuvant intensity-modulated radiotherapy (IMRT).

**Methods:**

Chemotherapy regimens included Cisplatin/Pemetrexed, Carboplatin/Pemetrexed and Cisplatin/Gemcitabine, followed by EPP. 62 patients completed the adjuvant radiotherapy. IMRT was carried out in two techniques, either step&shoot or helical tomotherapy. Median target dose was 48 Gy to 54 Gy. Toxicity was scored with the Common Terminology Criteria (CTC) for Adverse Events. We used Kaplan-Meier method to estimate actuarial rate of locoregional control (LRC), distant control (DC) and overall survival (OS), measured from the date of surgery. Rates were compared using the logrank test. For multivariate analysis the Cox proportional hazard model was used.

**Results:**

The median OS, LRC and DC times were 20.4, 31.4 and 21.4 months. The 1-, 2-, 3-year OS rates were 63, 42, 28 %, the LRC rates were 81, 60, 40 %, and the DC rates were 62, 48, 41 %. We observed no CTC grade 4 or grade 5 toxicity. Step&shoot and helical tomotherapy were equivalent both in dosimetric characteristics and clinical outcome. Biphasic tumor histology was associated with worse clinical outcome compared to epitheloid histology.

**Conclusions:**

Mature clinical results of trimodal treatment for MPM were presented. They indicate that hemithoracic radiotherapy after EPP can be safely administered by either step&shoot IMRT and tomotherapy. However, the optimal prospective patient selection for this aggressive trimodal therapy approach remains unclear. This study can serve as a benchmark for current and future therapy concepts for MPM.

**Electronic supplementary material:**

The online version of this article (doi:10.1186/s13014-015-0575-5) contains supplementary material, which is available to authorized users.

## Introduction

Malignant pleural mesothelioma (MPM) is an aggressive disease with a poor prognosis, currently treated with different combinations of chemotherapy, surgery and radiotherapy. For each single treatment modality, significant improvements have been achieved over the last years. With respect to chemotherapy regimens, Pemetrexed combined with Cisplatin has been established [1]; in surgery approaches, extrapleural pneumonectomy (EPP) and pleurectomy/decortication (P/D) have been technically advanced resulting in tolerable toxicity [2]; moreover the introduction of intensity-modulated radiotherapy (IMRT) allows to conform the high dose area tightly to the target volume and spare adjacent organs at risk [3].

However, despite these individual advancements the optimal combination and treatment strategy for each individual patient still remains unclear. Randomized clinical trials have been difficult to organize due to the rareness of the disease, patients presenting very differently at the time of diagnosis in terms of tumor spread, histology and general performance status, and different technical possibilities and experiences at different clinical centers. Therefore, evaluations of the clinical outcome of cohorts treated systematically by a certain regimen are relevant to assess its potential and also the associated risks for the patients.

In this work, we assessed and analyzed the clinical outcome of consecutive MPM patients treated in Heidelberg, Germany, by hemithoracic intensity-modulated radiotherapy in a trimodal setting after neoadjuvant chemotherapy and EPP.

## Methods

### Inclusion criteria

All MPM patients that completed adjuvant radiotherapy at the Heidelberg University Hospital after chemotherapy and EPP between 2003 and 2010 were included in this study. This required adequate recovery from surgery. To be eligible for EPP, the patients were required to have localized disease (maximum cT4N2M0), epitheloid or biphasic tumor histology, and a sufficient general performance status. This study was approved by the independent ethics committee of the medical faculty of the University of Heidelberg.

### Chemotherapy

All patients received neoadjuvant chemotherapy prior to surgery, consisting of either Cisplatin/Pemetrexed, Carboplatin/Pemetrexed or Cisplatin/Gemcitabine.

### Extrapleural pneumonectomy (EPP)

EPP included the removal of the complete afflicted lung together with the parietal pleura, pericardium and diaphragm. It was performed via an extended S-shaped anterolateral thoracotomy incision in the sixth intercostal space. Sites of prior open biopsy, thoracoscopy incisions, or chest tube tracks were excised separately. After resection, the diaphragm was replaced by a Monofilament Polypropylene mesh (Bard Mesh; Davol, Inc, Cranston, RI), and the pericardium was reconstructed with a xenopericard patch (Supple Peri-Guard; Synovis Surgical Innovations, St Paul, MN). In all patients undergoing EPP, a systematic mediastinal lymph node dissection was performed.

### Radiotherapy

#### Immobilization

During the acquisition of the contrast-enhanced computed tomography (CT) scan for treatment planning (and also later for irradiation), patients were immobilized by a vacuum body mattress and a Scotch-cast head mask, with the arms above the head on a resting plate. The surgical incisions were marked by thin metal wires during the planning CT scan.

#### Segmentation

As planning target volume (PTV), the complete ipsilateral thoracic cavity was segmented from lung apex to insertion of the diaphragm, including the ribs, the reconstructed pericardium and diaphragm, and extending up to the skin surface along the surgical incisions, with a safety margin of 5 mm which could be reduced at directly adjacent organs at risk. It was also made sure that all internal metal clips from surgery were included in the target volume. The contralateral lung, heart, liver, esophagus, spinal cord, and kidneys were segmented as organs at risk.

#### IMRT planning

**Fig. 1:**
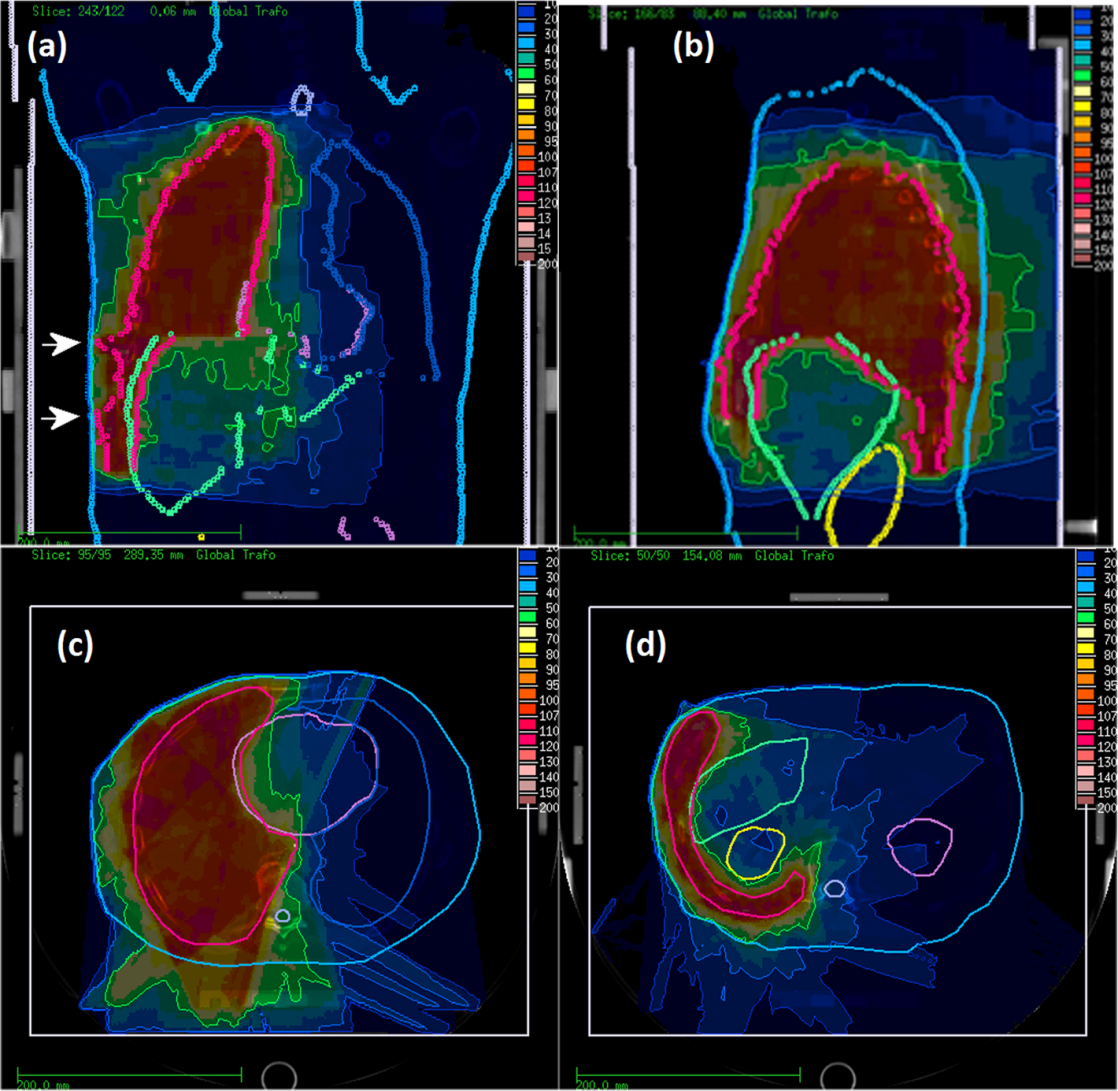
Structure definition and dose distribution for one MPM patient (Step & Shoot IMRT). In the coronar projection (**a**), the inclusion of the EPP incisions into the target volume are marked by white arrows. The sagittal projection (**b**) shows the caudal extension of the target volume with sparing of the liver and the kidney. In the two transverse slices the sparing of the heart (**c**) and again liver and kidney (**d**) can be seen

The treatment was performed either as step&shoot IMRT or helical tomotherapy IMRT. For step&shoot IMRT, treatment planning was performed using Konrad (Siemens, Erlangen, Germany). We placed 9–12 coplanar, non-equidistant beams in order to achieve optimal target coverage and sparing of the contralateral lung. For helical tomotherapy, we used the Tomotherapy planning system (Accuray, Sunnyvale, CA, USA). A field width of 2.5 cm was chosen. An example plan used for step-and-shoot IMRT treatment showing both segmentation and the resulting dose distribution is shown in Fig. 1.

#### IMRT delivery

Step&shoot IMRT was delivered by Siemens Primus and Siemens Artiste linear accelerators (Siemens, Erlangen, Germany) at 6 MV with regular image guidance (at least once per week) by an in-room CT scanner (Siemens Primatom, Erlangen, Germany). Helical tomotherapy was delivered by a Tomotherapy Hi-Art system (Accuray, Sunnyvale, CA, USA) at 6 MV, using built-in image guidance acquired at 3 MV. Median target dose was 48 to 54 Gy in 2 Gy fractions.

### Follow-up

Regular follow up visits were performed at our institution or the referring center. At our institution, patients were scheduled for follow up visits 6 weeks after end of radiotherapy and then every 3 months for the first 2 years, every 6 months for the three following years and annually thereafter. Each follow-up visit included a patient interview, clinical examination and computed tomography (CT) of the chest. In case of evidence for locoregional recurrence or distant spread, additional tests or imaging studies were performed to confirm or exclude disease progression at the discretion of the treating physician. Missing data were completed by calling the patient and/or the treating physician.

Toxicity was scored with the Common Terminology Criteria (CTC) for Adverse Events version 4.0. Locoregional failure was defined as tumor relapse within the treated ipsilateral hemithorax. Distant control was defined as absence of tumor relapse outside the treated ipsilateral hemithorax. In patients without assessment of locoregional control/distant spread at the time of death, the timepoint of the last locoregional control/distant spread status was used for calculation.

### Statistical analysis

The Kaplan-Meier method was used to estimate actuarial rates of locoregional control (LRC), distant control (DC), and overall survival (OS). Subgroups were compared using the logrank test. The Cox proportional hazard model was used for multivariate analysis. A *p*-value of ≤ 0.05 was considered significant. Survival times were calculated from the date of EPP surgery. Data were analysed using Statistica 6.0 (Statsoft, Tulsa, OK, USA).

## Results

### Patient characteristics

Between 2003 and 2010, 62 patients (pts) completed postoperative radiotherapy after EPP in a trimodal treatment setting in our institution. The patient characteristics are outlined in Table 1. The most common postoperative tumor staging was ypT3N0M0 (with 41 pts T3 and 43 pts N0), but also T2 (7 pts), T4 (4 pts), N1 (7 pts) and N2 (6 pts) stages occurred.

**Table 1:** Patient characteristics

Total number of patients	62
Age at diagnosis	
Median	57.9 years
Range	[34.5–70.4 years]
Gender	
Male	52 (83.9 %)
Female	10 (16.1 %)
Tumor location	
Left	27 (43.5 %)
Right	35 (56.5 %)
Histology	
Epitheloid	44 (70.9 %)
Biphasic	18 (29.1 %)

### Treatment characteristics

All patients received neoadjuvant chemotherapy. Regimens used were Cisplatin/Pemetrexed (30 pts, 48.4 %), Carboplatin/Pemetrexed (23 pts, 37.1 %) and Cisplatin/Gemcitabine (9 pts, 14.5 %). Mostly 3 or 4 cycles of chemotherapy were given; 5 patients received 6 cycles and 1 patient received 7 cycles. The EPP surgery was carried out mainly at the Thoracic Hospital at the University Clinic Heidelberg (58 pts, 82.9 %), but also in other hospitals (12 pts, 17.1 %). The median interval between the date of diagnosis and date of EPP surgery was 4.4 months. The median interval between surgery and start of radiotherapy was 8.4 weeks (range, 1.3–46.1 weeks).

We started intensity-modulated radiotherapy for MPM in 2003 with step & shoot technique. From 2006 on, helical tomotherapy was used as an additional IMRT technique. The first patients received median doses of 48Gy (2 pts, 3.2 %) and 50Gy (16 pts, 25.8 %) to the target volume in 2 Gy fractions. Later, the median target dose was increased to 54Gy (44 pts, 71.0 %). In total, 41 pts (66.1 %) were treated with step&shoot and 21 pts (33.9 %) with helical tomotherapy IMRT.

### Dosimetric comparison of step&shoot and helical tomotherapy IMRT

Several key dosimetric parameters were extracted from the treatment planning systems to characterize the treatment. They are listed in Table 2, including the standard deviation (SD). The parameters are listed separately for step&shoot-IMRT and helical tomotherapy as well as combined for all patients. The mean target dose is a little lower for step&shoot, since some of these patients were planned to a median target dose of 48–50Gy, whereas all tomotherapy patients were planned to a median target dose of 54Gy. The target dose coverage and homogeneity is slightly better for helical tomotherapy, with doses to some organs at risk (liver for right-sided MPM, heart) also slightly higher. Especially the contralateral lung could be spared very effectively with a mean lung dose (MLD) of (7.6 ± 2.2) Gy, a V5Gy of (66.2 ± 23.0) % and a V20Gy of (1.7 ± 1.9) % for step&shoot-IMRT and a MLD of (7.0 ± 1.2) Gy, a V5Gy of (71.5 ± 18.6) % and a V20Gy of (0.7 ± 1.3) % for helical tomotherapy, respectively.

**Table 2:** Dosimetric parameters of the treatment plans

	Step & Shoot	Helical Tomotherapy	All Patients
	41 patients	21 patients	62 patients
	Mean value ± SD	Mean value ± SD	Mean value ± SD
Target volume (PTV)			
Mean Dose [Gy]	52.1 ± 2.3	53.5 ± 0.5	52.6 ± 2.0
Standard Deviation [Gy]	3.5 ± 1.5	2.4 ± 1.2	3.1 ± 1.5
V95% [%]	85.3 ± 5.3	92.3 ± 5.2	87.6 ± 6.2
V90% [%]	93.3 ± 3.8	94.2 ± 3.9	94.2 ± 3.9
Contralateral Lung			
Mean Lung Dose [Gy]	7.6 ± 2.2	7.0 ± 1.2	7.4 ± 2.0
V5Gy [%]	66.2 ± 23.0	71.5 ± 18.6	67.9 ± 21.6
V20Gy [%]	1.7 ± 1.9	0.7 ± 1.3	1.4 ± 1.8
Liver (right-sided MPM)			
Mean Dose [Gy]	21.9 ± 3.9	26.4 ± 3.8	23.4 ± 4.4
V30Gy [%]	24.7 ± 8.3	33.5 ± 7.8	27.5 ± 9.0
Liver (left-sided MPM)			
Mean Dose [Gy]	9.1 ± 2.3	10.4 ± 1.5	9.6 ± 2.1
V30Gy [%]	2.0 ± 2.5	2.5 ± 2.7	2.2 ± 2.5
Heart			
V45Gy [%]	7.3 ± 7.5	12.4 ± 6.1	9.0 ± 7.4
Ipsilateral kidney			
Mean Dose [Gy]	9.9 ± 5.1	9.3 ± 2.5	9.7 ± 4.4
V15Gy [%]	23.4 ± 23.2	16.5 ± 13.0	21.0 ± 20.4
Contralateral kidney			
Mean Dose [Gy]	3.1 ± 1.6	4.5 ± 1.5	3.6 ± 1.7
V15Gy [%]	0.1 ± 0.2	0.3 ± 0.6	0.2 ± 0.4
Spinal Cord			
Maximum Dose [Gy]	36.0 ± 5.7	37.3 ± 5.3	36.4 ± 5.5
Esophagus			
V55Gy [%]	0.9 ± 1.7	2.2 ± 2.4	1.4 ± 2.1

*SD* standard deviation, *PTV* planning target volume

### Toxicity of radiotherapy

Most common side effects were mild nausea and skin erythema CTC grade 1–2. Many patients reported fatigue during radiation treatment which improved after completion of treatment. One patient developed symptomatic anaemia during therapy, requiring the transfusion of erythrocyte concentrates, but recovered well. One patient showed radiological signs of pneumonitis in the CT scan after radiotherapy, but was clinically asymptomatic and needed no intervention (pneumonitis CTC grade 1). One patient (treated with step&shoot IMRT, MLD 10.7 Gy, V5Gy 92 %, V20Gy 5 %) developed clinically symptomatic radiation pneumonitis, which resolved completely after treatment with prednisolone (CTC grade 3). We observed no CTC grade 4 or grade 5 adverse effects of the irradiation.

### Overall survival, locoregional control, distant control

The median follow-up time was 17.0 months (range, 2.4–111.9 months). Only 6 patients were still alive at the time of analysis, with a median follow-up time of 60.6 months (range, 8.9–111.9 months). The data point at 8.9 months was censored because the patient was lost to follow-up, the other patients alive at the time of analysis had a follow-up time of at least 40 months.

The median overall survival (OS) for all 62 pts was 20.4 months. The OS after 1 year, 2 years and 3 years was 63 % (SD 6 %), 42 % (SD 6 %) and 28 % (SD 6 %), respectively. Median locoregional control (LRC) was 31.4 months. LRC after 1 year, 2 years and 3 years was 81 % (SD 6 %), 60 % (SD 9 %) and 40 % (SD 11 %), respectively. Median distant control (DC) was 21.4 months. DC after 1 year, 2 years and 3 years was 62 % (SD 7 %), 48 % (SD 8 %) and 41 % (SD 10 %), respectively.

**Fig. 2:**
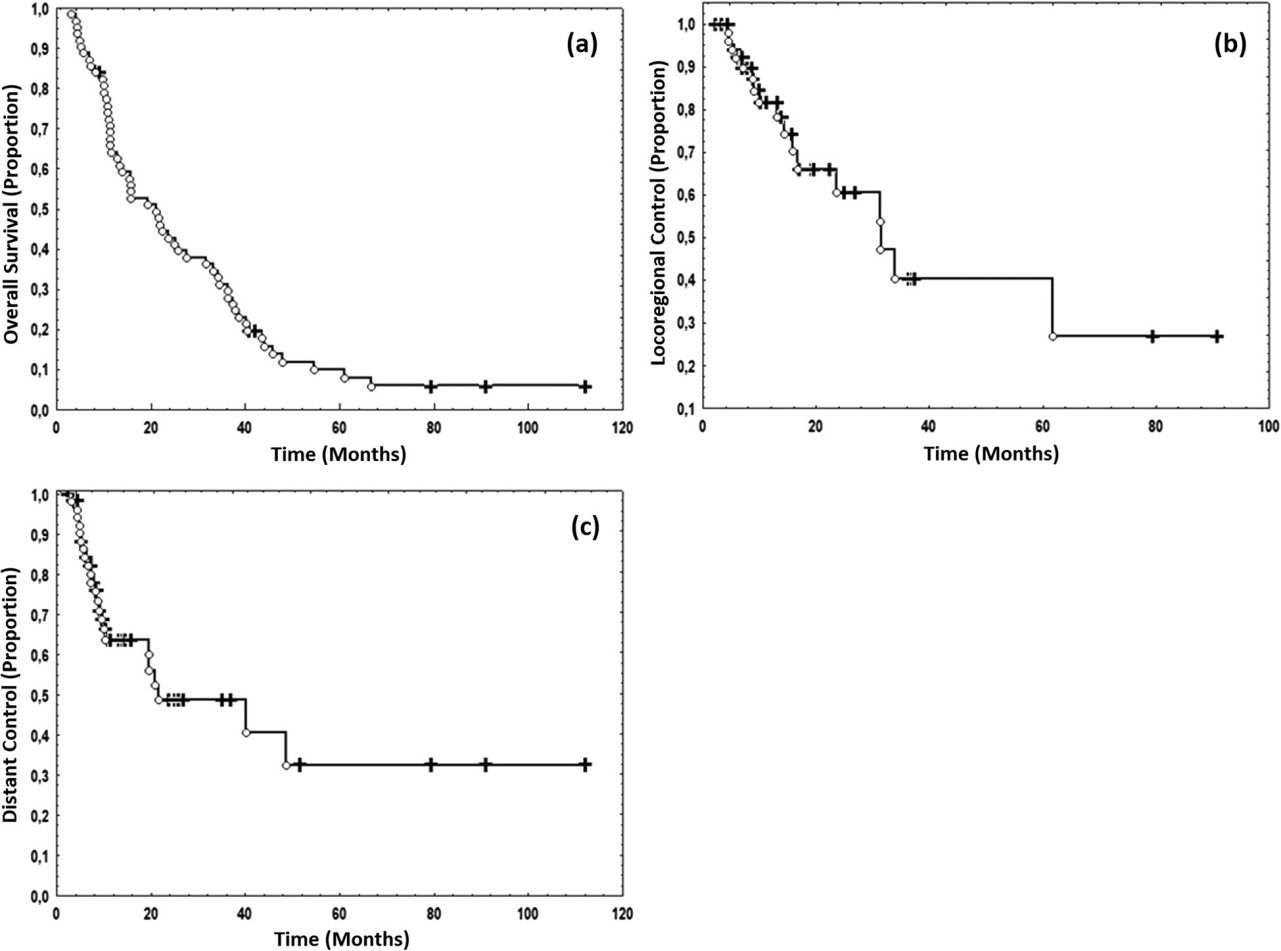
Kaplan-Meier curves for all patients. **a** Overall survival, **b** locoregional control and **c** distant control

The Kaplan-Meier curves for OS, LRC and DC are shown in Fig. 2.

### Factors associated with survival

**Fig. 3:**
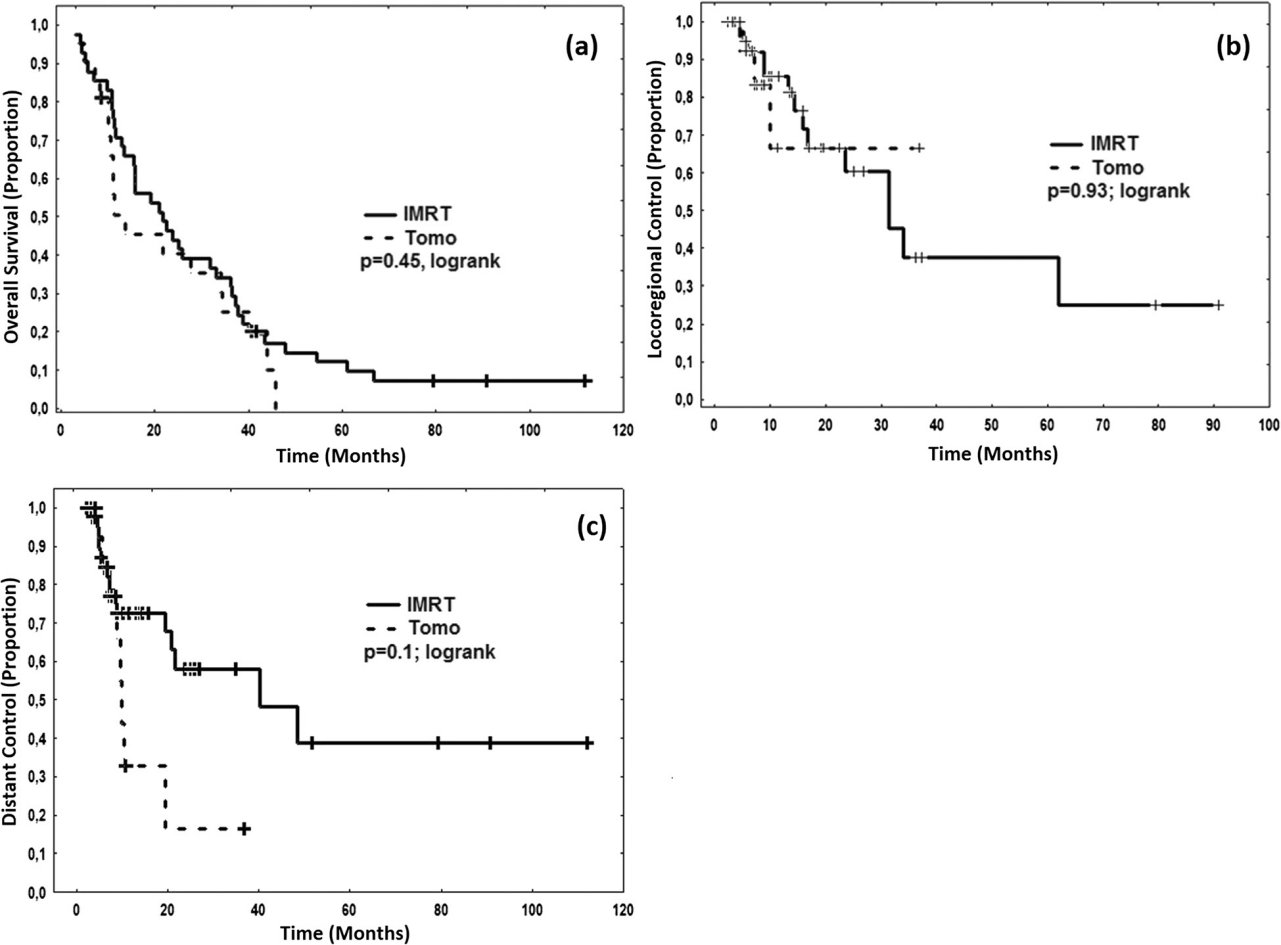
Kaplan-Meier curves separated for type of radiation treatment. Step&shoot-IMRT (“IMRT”) vs. helical tomotherapy-IMRT (“Tomo”) shows no significant difference regarding **a** overall survival, **b** locoregional control and **c** distant control

**Fig. 4:**
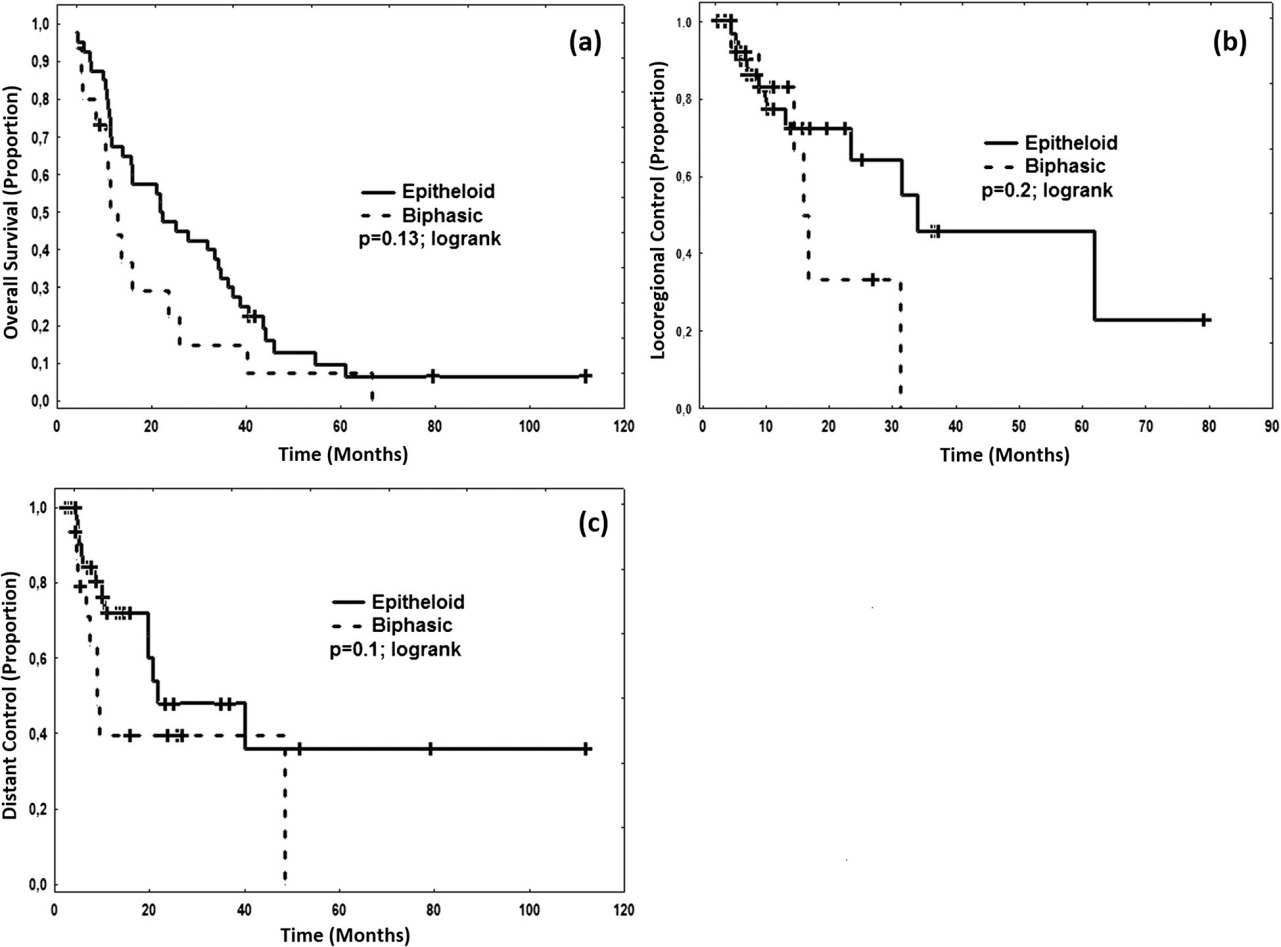
Kaplan-Meier curves separated for histology. Biphasic histology tended to be associated with worse prognosis compared to epitheloid histology with respect to **a** overall survival, **b** locoregional control and **c** distant control

Multivariate analysis showed that with respect to OS among all the variables tested, the male gender tended to result in worse prognosis although not reaching significance (Hazard Ratio (HR) 1.7; 95 % Confidence Interval (CI95) 0.7–4.9; *p* = 0.2). The only two significant variables were the postoperative resection status R (per higher status HR 3.9; CI95 1.3–12.2; *p* = 0.01 and biphasic histology (HR 2.2; CI95 1.2–5. 4; *p* = 0.03). With respect to locoregional control, no variable tested reached significance in multivariate analysis. However higher R status (HR 3.2; CI95 0.45–23.5; *p* = 0.2) and biphasic histology (HR = 3.4; CI95 0.77–14. 9; *p* = 0.1) tended to result in reduced local control. With respect to distant control, both higher R Status (HR 10.9; CI95 1.3–76. 2; *p* = 0.02) and biphasic histology (HR 7.4; CI95 2.2–25.4; *p* = 0.003) were significantly associated with worse outcome. Other variables including the IMRT technique (step&shoot vs. helical tomotherapy), dose distributions such as target mean dose, target coverage, lung dose, clinical factors such as lymph node involvement (N status), and patient factors such as age had no influence on OS, LRC and DC. Kaplan-Meier curves illustrating the equivalence of irradiation techniques are depicted in Fig. 3 and the influence of the histology is depicted in Fig. 4.

## Discussion

In this work we present the long-term clinical outcome of trimodal therapy of MPM consisting of neoadjuvant chemotherapy, EPP and adjuvant hemithoracic IMRT from a single institution. To our knowledge, our cohort of 62 consecutive patients is one of the largest with the longest follow-up times reported in the literature. The median OS, LRC and DC were 20.4, 31.4 and 21.4 months. The actuarial 1-, 2-, and 3-year OS was 63, 42, 28 %, the LRC 81, 60, 40 %, and the DC was 62, 48, 41 %. No CTC grade 4 or grade 5 toxicity was observed.

A cohort of 86 MPM patients treated with IMRT after EPP (57/86 without chemotherapy) were recently analyzed by Gomez et al. [4], who reported excellent locoregional control rates. Compared to these results, we observed longer median OS (20.4 vs. 14.7 months) with slightly better OS rates after 1 and 2 years (63 % vs. 55 % and 42 % vs. 32 %). Our locoregional control rates after 1 and 2 years were slightly worse (81 % vs. 88 % and 60 % vs. 71 %), but distant control rates after 1 and 2 years slightly better (62 % vs. 55 % and 48 % vs. 40 %). The dosimetric parameters of the radiotherapy were roughly equivalent. The chemotherapy might have caused the differences in DC and OS. This is consistent with other reports where trimodal treatment of MPM led to survival times of 20 months or more [5, 6]. We have summarized the treatment characteristics, survival times and acute lung toxicity of several reports in a table that is accompanying this paper as an additional file (see “Additional file 1”).

As reference time point from which the survival times were measured, we chose the date of surgery, as in [4, 7, 8]. This has to be considered when comparing the results with other reference time points such as the date of diagnosis [9–11], start of chemotherapy [12] or date of study enrolment [13] to avoid a lead time bias of up to several months (in our case, a median of 4.4 months between diagnosis and the day of EPP surgery). The survival times we observed were among the best reported in the recent medical literature [4, 7–17].

One main organ system to be considered regarding the toxicity of radiotherapy is the remaining contralateral lung. The radiotherapy, even delivered up to median target doses of 54 Gy, was well tolerated with no higher (4 or 5) grade toxicity to the contralateral lung. The mean lung dose (MLD) to the contralateral lung was (7.4 ± 2.0) Gy in all patients. Considering the rate of fatal pneumonitis reported several years ago (6 out of 13 patients experiencing grade 5 pneumonitis, with a median MLD of 15.2 Gy in the group of patients developing pneumonitis [18]) and the improvements made over the last years regarding better lung sparing, resulting in lower lung toxicity [7], there seems to be a dose-effect relationship for fatal pneumonitis, and an MLD around 7 Gy seems to be associated with an acceptable low risk of higher grade lung toxicity [8]. Because of the severe and fatal adverse effects of radiotherapy reported in other cohorts, we think that even though we did not observe high grade toxicity, the radiotherapy protocol should not be intensified further, neither by enlargening the target volume nor by escalating the dose beyond 54 Gy, unless new radiotherapy techniques will become available that would allow such intensification without increasing the dose to the organs at risk. Since all patients received IMRT with image guidance, using inverse planning and collimators with narrow leafs, the radiotherapy the patients received can be considered as state-of-the-art even for today’s standards.

Comparing the two IMRT techniques step&shoot and helical tomotherapy, we found no significant difference, neither in dosimetric terms (see also [19]) nor in clinical outcome. This notion is also supported by the finding that none of the dosimetric variables that were statistically analyzed, including the median target dose, affected clinical outcome such as OS, LRC and DC. The results indicate that both techniques nowadays allow for safe conformal hemithoracic radiotherapy with effective sparing of organs at risk, especially the contralateral lung. Major physical parameters for step&shoot IMRT and helical tomotherapy were similar, explaining the equivalence in dosimetric terms and clinical outcome: The leaf width of the multileaf colimator (MLC) in the isocenter plane was 5 mm for step&shoot and 6.25 mm for helical tomotherapy, both accelerators operated at 6 MV, and both techniques included image guidance (in-room CT for step&shoot, integrated MV-CT for tomotherapy).

Further improvements in radiotherapy might be achieved in the future in particular with respect to side effect reduction, e.g., through volumetric rotational irradiation (e.g., [20]) or through the use of particles. These techniques will require also higher standards regarding plan robustness, image guidance and plan adaptation.

One major point of discussion and controversy in clinical treatment of MPM is the radicality and aggressiveness, respectively, of the therapy concept, mainly determined by the surgical procedure. It has been argued that EPP can do more harm than good because of its peri- and postoperative morbidity and mortality [21], and that even chemotherapy alone can result in similar survival times [22]. However the maturity of the clinical data or the patient numbers are often limited, or other factors make comparisons difficult, so that there are no definite conclusions possible yet. E.g., the MARS study [21] was a feasibility trial in which only 19 patients actually received EPP. In [22] the median OS of the whole cohort was 13 months while only a subgroup of 51 out of 173 patients reached 22 months, and the authors state that comparisons with other studies are difficult because surgery for MPM in Scandinavia, where the study was conducted, is performed only for very early stages with the best performance status.

In recent years, the less radical pleurectomy/decortication (P/D) surgery procedure has frequently been proposed and carried out as an alternative to EPP surgery. In a systematic review and meta-analysis [2], it was stated that P/D might be performed with lower morbidity and mortality than EPP while resulting in comparable long-term survival, however the authors also noted that the comparison of both procedures has several limitations and the choice for a specific therapy is still highly individual based on the extension of the disease, the patient comorbidities and the center’s experience. The dose-shaping potential of IMRT allows high-dose (50 Gy) adjuvant radiotherapy even after P/D where the ipsilateral lung has to be spared as an additional organ at risk. First reports indicate promising clinical results: A median OS of 24 months (from date of diagnosis, 76 pts [23]), 33 months (from date of surgery, 20 pts [24]) and 28.4 months (from date of surgery, 24 pts [25]). The follow-up times however are still somewhat limited.

We carried out a retrospective study on a group of MPM patients selected in the sense that they were able to receive adjuvant radiotherapy after chemotherapy and EPP. The decision for radiotherapy was made after EPP individually for each patient depending on the postoperative development, so there was no strict intention-to-treat for trimodal therapy from the beginning. EPPs were performed in several different hospitals with some patients being referred to our institution for radiotherapy only. Therefore, an analysis that included also the patients having received EPP without adjuvant radiotherapy was not possible. Given these limitations, and the fact that the disease is relatively rare and no uniform treatment technique and algorithm has been established, our results, similar to published results by other groups, can only be compared with historical data.

Clearly, randomized clinical trials would be ideal for investigating the optimal therapy regimen for each individual patient, but as described, they are hard to conduct. Therefore the main findings of the presented study are 1) the conclusions regarding the safety of the adjuvant radiotherapy after EPP in general, 2) the shown equivalence of step&shoot IMRT and helical tomotherapy and 3) the mature clinical outcome data for trimodal therapy. We believe that the data may contribute to some guidance what can be optimally expected from this complicated, aggressive and technically sophisticated regimen. Furthermore, the data can serve as a benchmark to be compared to in future reports on the clinical outcome of other treatment strategies.

MPM remains a therapeutic challenge. Despite the radical strategy, the overall survival times presented here still show the poor prognosis of MPM patients. Both the rate of distant metastases and local treatments need further improvements by systemic and local options. While surgery, radiotherapy and conventional chemotherapy will probably remain the stage dependent mainstay of MPM therapy, it is likely that future concepts might integrate novel strategies such as targeted drugs or immunological approaches based on personalized molecular medicine data.

## Conclusions

Mature clinical results of trimodal treatment for MPM were presented. They indicate that hemithoracic radiotherapy after EPP can be safely administered by either step&shoot IMRT and tomotherapy. However, the optimal prospective patient selection for this aggressive trimodal therapy approach remains unclear. This study can serve as a benchmark for current and future therapy concepts for MPM.

## Additional file

Additional file 1:
**Literature overview of recent studies evaluating radiotherapy (RT) after extrapleural pneumonectomy (EPP).** (PDF 77 kb)
