# Supplementary material for: Long-term results in malignant pleural mesothelioma treated with neoadjuvant chemotherapy, extrapleural pneumonectomy and intensity-modulated radiotherapy
Source: Radiat Oncol. 2015 Dec 30;10:267. doi: 10.1186/s13014-015-0575-5 (PMC4696301; doi:10.1186/s13014-015-0575-5)
Supplement: Additional file 1: — Literature overview of recent studies evaluating radiotherapy (RT) after extrapleural pneumonectomy (EPP). (PDF 77 kb) [file 13014_2015_575_MOESM1_ESM.pdf]

Supplementary File 1:
Literature overview of recent studies evaluating radiotherapy (RT) after extrapleural pneumonectomy (EPP)

**Author (Institution)**	**RT Dose, Technique**	**Patients**	**Survival times**	**Acute RT Lung Toxicity**
Krayenbuehl et al. 2014 [12] (Zürich)	3D-CRT: 45Gy+12.6 Gy Boost (57.6 Gy)	25	From start of chemotherapy:<br><br>Local in-field recurrence rate 48%, marginal miss rate 4%. Median time to local relapse 10.9±5.4 months (72.7%), Median time to distant met. 16.7±7.7 months (80%). Median OS 26.9±11.8 months.	Not reported.
	IMRT/VMAT: 45.5Gy with SIB up to 55.9Gy	14	From start of chemotherapy:<br><br>Local in-field recurrence rate 21.4%, marginal miss rate 7.1%. Median time to distant met. 18.4±10.7 months (71.4%).<br>Median OS 20.8±14.4 months.	Not reported.
Rice et al. 2007 [14] (MD Anderson)<br><br>Gomez et al. 2013 [4] (MD Anderson)	IMRT, Median dose 45Gy, Boost up to 60Gy<br><br>IMRT	63 (for OS analysis) 61 (for recurrence analysis)<br><br>86	From date of surgery:<br><br>Locoregional recurrence rate 13%, local in-field recurrence rate 5%. Median OS 14.2 months, 3-year-OS 20%.<br><br>Median OS 14.7 months.<br>1-yr-OS 55%, 2-yr-OS 32%.<br>1-yr-LRC 88%, 2-yr-LRC 71%.<br>1-yr-DM-free 55%,2-yr-DM-free 40%.	Two grade 5 pneumonitis, four grade 5 pneumonia<br><br>Five grade 5 pulmonary toxicity
Stahel et al. 2014 [15] (Switzerland, SAKK 17/04)	CTV1 45/46Gy, CTV2 up to 55.9/56.2 Gy	27 started RT, 25 completed RT	Reference time point not specified in abstract.<br><br>Median local relapse-free survival 9.4 months (95% CI: 6.5-11.9).<br>Overall relapse-free survival 7.6 months (95% CI: 5.2-10.6).<br>OS 14.9 months (95% CI: 7.0-17.6).	Not reported (late-breaking abstract)

Sylvestre et al. 2011 [9], updated in Helou et al. 2013 [10] (Paris, Nantes)	Helical tomotherapy, median dose 50Gy	24 (29 in update)	From date of diagnosis:<br><br>2-yr disease-free survival 51.8% (95% CI: 30.0-90.0 months).<br>In update: 1yr-OS 65%, 2yr-OS 36%, median OS 18 months.	Two grade 5 pneumonitis suspected (median lung dose 11Gy).
Patel et al. 2012 [7] (Duke)	45Gy + 10-15Gy boost	30	From date of surgery:<br>Median OS 23.2 months.<br>2-yr-LC 47%, 2yr-DFS 34%, 2-yr-OS 50%.	No fatal lung toxicity in the last 15 pts. (Miles et al. 2008 reported one grade 5 pneumonitis)
Tonoli et al. 2011 [8] (Brescia, Florence, Modena)	3DCRT (4), IMRT (50), helical tomotherapy (2).<br>45-50Gy, boost up to 60Gy	56	From date of surgery:<br><br>3yr-locoregional control 90%.<br>3yr-distant met. Free survival 66%.<br>3yr-disease free survival 57%.<br>3yr-overall survival 60%.	No fatal lung toxicity.
Kristensen et al. 2009 [16] (Copenhagen)	IMRT.<br>50Gy, boost up to 60Gy	26	Not reported.	Four grade 5 pneumonitis, median MLD 13.9Gy
Federico et al. 2013 [13] (Padua, Milan, Rome, Mestre)	Median dose 54Gy, later 50.4Gy	54 pts. enrolled, 32 started RT, 25 completed RT	From date of enrolment:<br><br>Median progression-free survival 8.6 months (95% CI: 6.3-14.4).<br>Median OS 15.5 months (95% CI: 11.0-NA)	Dose reduction after two grade 5 cardiopulmonary events.
van Schil et al. 2010 [17] (EORTC 08031)	3DCRT(24), IMRT (14).<br>54Gy	59 pts enrolled, 38 started RT, 37 completed RT	Reference time point not specified.<br>Median PFS: 13.9 months (95% CI: 10.9-17.2).<br>Median OS: 18.4 months (95% CI: 15.6-32.9)<br>Median OS of the 37 pts who completed RT: 33 months (median hardly reached)	Two grade 5 pneumonia.
Fahrner et al. 2012 [11] (Bern)	60-65Gy	24 pts started trimodal therapy, 16 completed RT	From date of diagnosis:<br><br>1-, 2-, and 3-year OS 71%, 28%, 10%.<br><br>Median OS 23.2 months (range 2.1-59.4)<br>1-year-PFS 47%, 5-year-PFS 6%. Median PFS 18.8 months (range 2.6-74.3)	Not reported.
Current study	Step&shoot IMRT (41), helical tomotherapy (21)<br><br>48-54 Gy	62	From date of surgery:<br>Median OS 20.4 months.<br><br>1-, 2-, and 3-year OS 63%, 42%, 28%.	One grade 1 pneumonitis, one grade 3 pneumonitis. No fatal lung toxicity.
